# Supplementary material for: Random Copolymers of Lysine and Isoleucine for Efficient mRNA Delivery
Source: Int J Mol Sci. 2022 May 11;23(10):5363. doi: 10.3390/ijms23105363 (PMC9140483; doi:10.3390/ijms23105363)
Supplement: Supplementary file 1 [file ijms-23-05363-s001.zip › ijms-1697935-supplementary.pdf]

International Journal of
Molecular Sciences

MDPI

Supplementary Materials

Random Copolymers of Lysine and Isoleucine for Efficient mRNA Delivery

Iuliia Pilipenko [1,2], Olga Korovkina [1], Nina Gubina [1], Viktoria Ekimova [3], Anastasia Ishutinova [3], Evgenia Korzhikova-Vlakh [1,4], Tatiana Tennikova [1] and Viktor Korzhikov-Vlakh [1,*]

1 Institute of Chemistry, St. Petersburg State University, Universitetskii pr. 26, Peterhof, 198504 St. Petersburg, Russia; pilipenko@scamt-itmo.ru (I.P.); olya_osipova_06_01@mail.ru (O.K.); nina.gubina2000@gmail.com (N.G.); vlakh@mail.ru (E.K.-V.); t.tennikova@spbu.ru (T.T.)
2 International Laboratory "Solution Chemistry of Advanced Materials and Technologies", ITMO University, Lomonosova St. 9, 191002 St. Petersburg, Russia
3 CJSC Biocad, ul. Svyazi., 34-A, Strelna, 198515 St. Petersburg, Russia; ekimova@biocad.ru (V.E.); ishutinova@biocad.ru (A.I.)
4 Institute of Macromolecular Compounds, Russian Academy of Sciences, Bolshoy pr. 31, 199004 St. Petersburg, Russia
* Correspondence: v.korzhikov-vlakh@spbu.ru

Citation: Pilipenko, I.; Korovkina, O.; Gubina, N.; Ekimova, V.; Ishutinova, A.; Korzhikova-Vlakh, E.; Tennikova, T.; Korzhikov-Vlakh, V. Random Copolymers of Lysine and Isoleucine for Efficient mRNA Delivery. *Int. J. Mol. Sci.* **2022**, *23*, 5363. https://doi.org/10.3390/ijms23105363

Academic Editor: Anna Maria Piras

Received: 9 April 2022
Accepted: 9 May 2022
Published: 11 May 2022

Publisher's Note: MDPI stays neutral with regard to jurisdictional claims in published maps and institutional affiliations.

Copyright: © 2022 by the authors. Licensee MDPI, Basel, Switzerland. This article is an open access article distributed under the terms and conditions of the Creative Commons Attribution (CC BY) license (http://creativecommons.org/licenses/by/4.0/).

Table S1. Synthesized polypeptides and particles characteristics, and their efficacy towards mRNA binding. Monomers to initiator ratio was maintained at 50. Polymer:mRNA mass ratio was maintained at 4/1.

Sample	[Lys]:[Ile] initial ratio	Composition of copolymer, mol% (HPLC [a])		SEC data [b]			DLS and ELS data [c]		mRNA binding efficacy, %
		Lys	*Ile*	*Mn*	*Mw*	$Đ_M$	D_H, *nm* ±SD [3]	*ζ-potential, mV* ±SD [4]	
P(Lys-co-Ile)	60:40	66.9	33.1	8.300	11.500	1.38	550±41	42±2	-
P(Lys-co-Ile) +mRNA	60:40	-“-	-“-	-“-	-“-	-“-	467±48	33±6	62
P(Lys-co-Ile)	70:30	77.6	22.4	7.300	9.500	1.29	510±43	38±5	-
P(Lys-co-Ile) +mRNA	70:30	-“-	-“-	-“-	-“-	-“-	405±28	30±6	77
P(Lys-co-Ile)	80:20	85.7	14.3	6.300	8.100	1.28	400±48	39±6	-
P(Lys-co-Ile) +mRNA	80:20	-“-	-“-	-“-	-“-	-“-	134±	29±7	95

[a] data were obtained for Z-protected polymers with application of PMMA standards; [b] data were obtained for the self-assembled polymer particles after removal of Z-protection; [c] particles were obtained by ultrasonication during 30 sec. Size and ζ-potential were measured in ultrapure water.

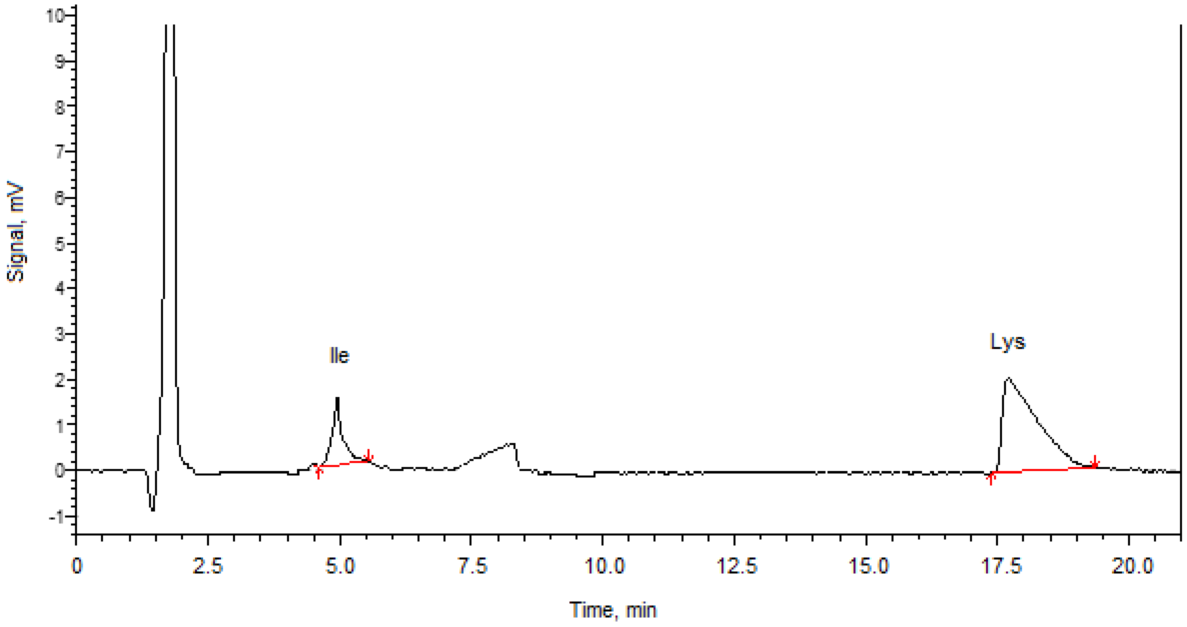

Figure S1. HPLC chromatogram obtained during analysis of hydrolysate of P(Lys-co-Ile) copolymer synthesized with initial [Lys]/[Ile] equal to 70/30. *Equipment and conditions*: LC-20 Prominence HPLC system (Shimadzu) equipped with the RID-20A refractometric detector and Shodex IC YS-50 column of 125 mm ×4.6 mm (5 µm beads). 3 mM H_3PO_4 was used as an eluent; flow rate was 0.8 mL/min, injection loop volume was 20 µL. Analysis was performed at 40 °C. Equations for calibration plots were Y = aX + b where for Ile: a = 557.066, b = 0, R^2 = 0.9996053, R = 0.9998027; for Lys: a = 672.324, b = 0, R^2 = 0.9992335, R = 0.9996167. Concentration ranges used for calibration were 0.5-20 µg/mL for Lys and 1-20 µg/mL for Ile.

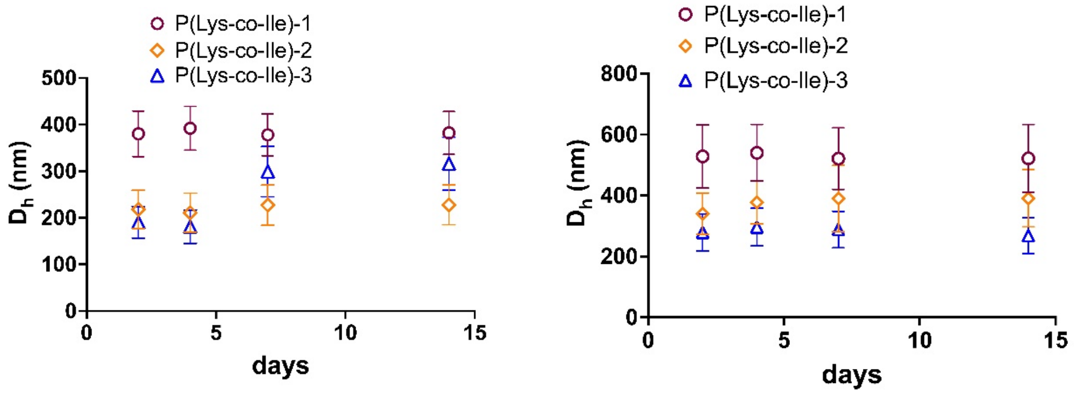

(**A**) (**B**)

Figure S2. The change of P(Lys-co-Ile) particles hydrodynamic diameter during two weeks: (A) incubation of particles in 0.01M PBS, pH 7.4; (B) incubation of particles in FBS-free Opti MEM cell culture medium.

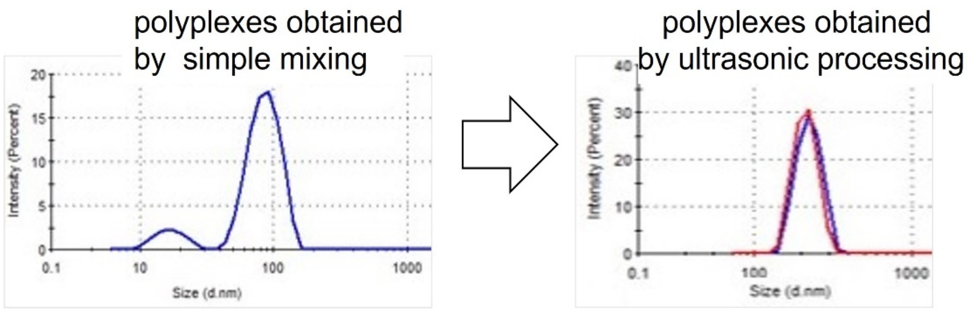

Figure S3. Effect of US on particles size distribution appearance. P(Lys-co-Ile)-3 copolymer polyplex with RNA/Ca^{2+} was applied here at N/P = 4. The measurements were performed in ultrapure H_2O.

Enzymatic stability of DNA

Pure DNA (Deoxyribonucleic Acid (DNA), Sodium Salt from Salmon Testes, Sigma Aldrich) and its complexes with cationic polypeptides (N/P = 4) were coincubated with enzyme (DNase I from Bovine Pancrease, Merck). The degradation and stability of DNA were detected by 1 % agarose gel electrophoresis (**Fig. 8**). Comparison of lanes 2 and 3 clearly shows that unprotected DNA is degraded by DNase. Complexation of DNA within polyplex based on P(Lys-co-Ile) at N/P 4 resulted in the stabilization of DNA towards degradation by DNase. Lane 5 (**Fig. 8**) shows the fate of DNA, which was released from polyplex under action of heparin excess. The released DNA was degraded by enzyme, which was present in the same mixture. Lane 6 (**Fig. 8**) shows the stability of DNA, which was released from polyplex by addition of heparin excess after DNase removal from the mixture. In this case, we can observe the same mobility of released DNA as that for initial one.

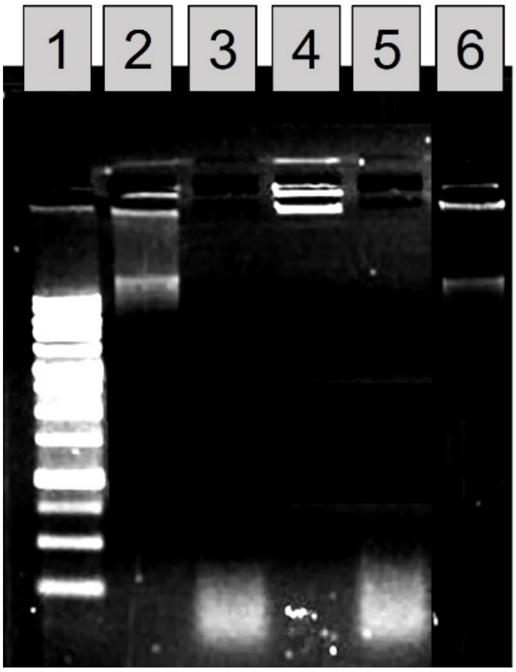

Figure S4. Stability of pDNA-luc within complexes with P(Lys-co-Ile) towards degradation by DNase I. Experiment was performed with P(Lys-co-Ile)-3 at N/P = 4. 100 V for 15 mins and 120 V for 45 mins. Lane 1 – 1 kb DNA Ladder; Lane 2 – DNA; Lane 3 – pDNA + DNase; Lane 4 – P(Lys-co-Ile)-3+ DNA + DNase; Lane 5 – P(Lys-co-Ile)-3+ DNA +heparin (P(Lys-co-Ile)/heparin = 8 w/w) + DNase; Lane 6 – P(Lys-co-Ile)-3+ DNA+ DNase + heparin (after removal of DNA; P(Lys-co-Ile)/heparin = 8 w/w).

Luciferase pDNA transfection

Cells were seeded at a density of 50,000 cells per well. Next day, the cell culture medium was refreshed and 50 µL of nanoparticles, containing 1 µg of pDNA-luc, varying the concentration of polymer according to the N/P ratio desired. The cells were incubated with nanoparticles in a serum-free medium for 4 h, then the medium was refreshed with a serum-containing medium. After 24 h a half of the supernatant (150 µL) was collected, and after 48 hour remaining 250 µL supernatant was collected for the analysis.

Analysis was performed using Gaussia Luciferase kit (Thermo Fisher Scientific). Briefly, 20 µL of each sample was transferred to 96-well plate (Black/White Corning) and mixed with 50 µL of substrate 1x coelenterazine, which was prepared in Gaussia Flash Assay Buffer. Luminescence was measured at 485/535 nm with application of Microplate Spectrophotometer-Fluorometer Fluoroskan Ascent reader. Transfection efficiency obtained by PEI/pDNA polyplexes was used as control.

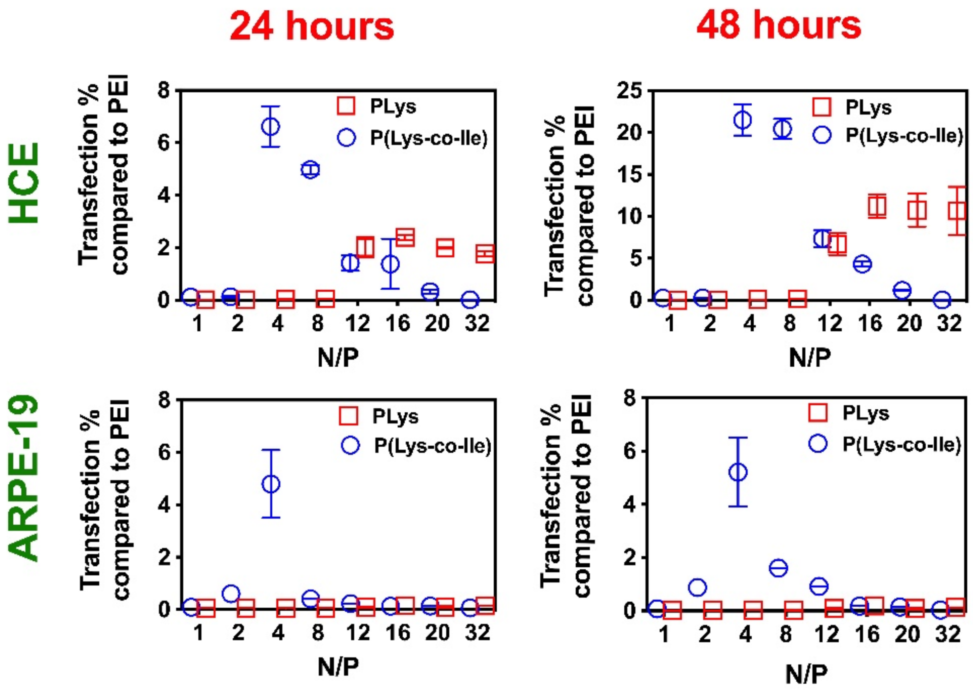

Figure S5. Transfection of HCE and ARPE-19 cells with pDNA-luc polyplexes with PLys and P(Lys-co-Ile)-3. Mean (±SD), n = 5.

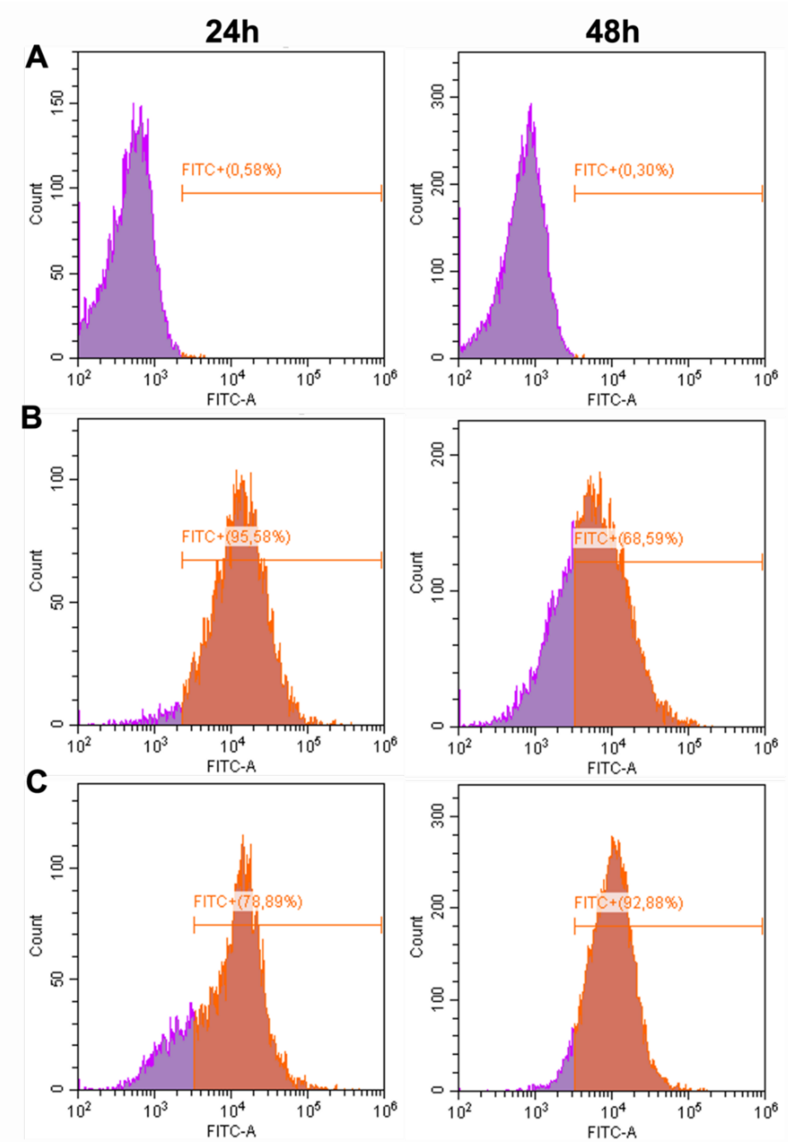

Figure S6. Transfection of K562 cell line with (A) EGFP-mRNA control; (**B**) Lipofectamine 2000-mRNA-GFP lipoplexes and (**C**) P(Lys-co-Ile)+EGFP-mRNA/Ca^{2+} N/P 4 polyplexes after 24 and 48 h post-transfection.

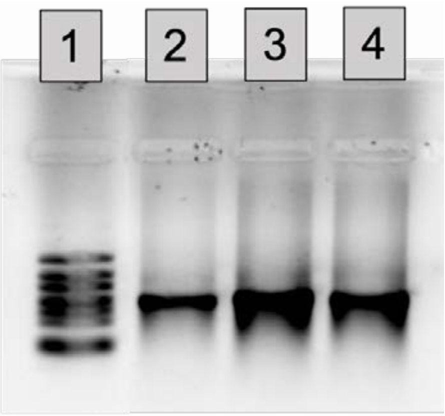

Figure S7. 1 % agarose denaturing gel electrophoresis, showing mRNA obtained from different vials after synthesis. Lane 1 – Ribo Ruller HighRange; Lane 2 – initial mRNA control from vial #1; Lane 3 – initial mRNA control from vial #2; Lane 4 – initial mRNA control from vial #3.
